# Factors influencing adherence to antiretroviral therapy among young adults in Limpopo province

**DOI:** 10.4102/safp.v66i1.5973

**Published:** 2024-08-05

**Authors:** Victoria Mashele, Gert J.O. Marincowitz, Clara Marincowitz

**Affiliations:** 1 Department of Family Medicine, Faculty of Health Sciences, University of Limpopo, Polokwane, South Africa; 2 Health Limpopo, Mankweng Hospital, Polokwane, South Africa; 3 Department of Family Medicine, Limpopo Department of Health, Mankweng Hospital, Polokwane, South Africa; 4 Department of Psychiatry, Faculty of Health Sciences, Stellenbosch University, Tygerberg, South Africa; 5 SA Medical Research Council, Cape Town, South Africa

**Keywords:** antiretroviral therapy, adherence, factors influencing, challenges to antiretroviral therapy

## Abstract

**Background:**

South Africa is among the countries with the greatest burden of human immunodeficiency virus (HIV) in the world. The introduction of antiretroviral therapy (ART) has made HIV a manageable chronic health condition with a return to normal life expectancy. Adherence to ART is a prerequisite to realising these benefits.

**Methods:**

A qualitative study was conducted using individual semi-structured interviews to understand factors influencing adherence to ART among young adults. The study was conducted at three busy primary care clinics around Mankweng Hospital. Participants aged 18–35 years who had been on ART for more than a year were purposefully selected. Open-ended questions were used to explore factors that influence ART. Recorded interviews were transcribed verbatim and translated. The coded transcripts were thematically analysed.

**Results:**

Eight major themes were identified to influence ART adherence among young adults: medication-related factors, healthcare system factors, attitudes of healthcare workers, economic factors, disclosure, acceptance, mobile phone reminders and family support.

**Conclusion:**

Adherence to ART is a major problem in our communities, and people living with HIV are still finding it challenging to optimally adhere to their ART medication because of the identified factors that influence ART adherence. Family support is a significant factor that was identified to positively influence ART as it leads to disclosure and acceptance of HIV-positive status, better emotional well-being and subsequently improved ART adherence.

**Contribution:**

This study underscores the importance of a family-oriented, patient-centred care approach in managing HIV and ART adherence.

## Introduction

Human immunodeficiency virus (HIV) continues to be a major public health challenge, with an estimated 39 million people living with HIV globally at the end of 2022. Of these people living with HIV, 23.3m are accessing antiretroviral therapy (ART).^1,2,3^ South Africa has one of the greatest burdens of HIV in the world, with 7.06m people living with HIV, of whom approximately 260 893 are in Limpopo province.^3^ Committed to attaining the Joint United Nations Programme on HIV/AIDS (UNAIDS) 95-95-95 targets to control the HIV epidemic, South Africa provides the most extensive public ART programme in the world, with over 80% of people living with HIV on ART.^2,4,5^

The introduction of ART has made HIV a manageable chronic health condition with a return to normal life expectancy and a better quality of life.^6,7,8^ However, adherence to ART is a prerequisite to realising these benefits.^6^ According to the World Health Organization (WHO), an adherence benchmark of 95% is required for sustained viral load suppression.^1^ Poor adherence to ART is a major cause of treatment failure, and it is associated with increased transmission rates, more opportunistic infections, drug resistance and cost implications.^5,8^ Adherence is described as how a person uses treatment according to medical recommendations, which includes timing, dosing and consistency.^8,9,10^

In the Limpopo province of South Africa, ART adherence was 87% among young adults.^11^ However, this study tested adherence in a self-reported questionnaire and excluded patients who stopped treatment and were lost to follow-up, possibly giving a biased impression of adherence. Conversely, the Heath Systems Trust District Barometer for 2022/2023 presented both the proportion of patients remaining on ART as well as the viral load suppression rates of patients on ART as indicators for the successful implementation of the ART programme.^12^ According to this report, the National South African proportion of patients living with HIV who are still on ART by March 2023 was 68.2%. For Limpopo province, this number was 8% lower at 60.4%, placing the province second to last. The National viral load suppression rate for patients who are actively on ART from age 15 years and older was 90%, with Limpopo province falling slightly worse at 89%. Patient adherence to ART is a crucial element of the successful implementation of the ART programme. Poor adherence leads to treatment failure and possible treatment resistance. Therefore, with only 60.4% of HIV patients who were still on ART and continuing care, the level of adherence in Limpopo province is clearly concerning.^12^

Antiretroviral therapy adherence is a complex and dynamic process with multiple challenges. Stigma and discrimination, family relationships, economics, treatment side effects, poor healthcare provider attitudes and health system inadequacies have all been shown to result in poor adherence.^6,7,8,13^ A South African-based cross-sectional survey also found that the prevalence of HIV infection is highest in young adults, and they have their lives ahead of them to be on medication to control the disease.^14^ Understanding factors that influence adherence in this group of patients is crucial to developing strategies for addressing their long-term treatment journey.

The authors therefore conducted a qualitative investigation to identify factors influencing adherence to ART among young adults living with HIV attending clinics around Mankweng Hospital in the Limpopo province of South Africa.

## Research methods and design

This qualitative study comprised semi-structured interviews with young adults receiving their ART at primary care clinics around Mankweng Hospital. The hospital is situated 30 km east of Polokwane town in Limpopo province, South Africa, and it is a tertiary and academic hospital that is affiliated with the University of Limpopo. Purposive sampling was used to select young adults between 18 years and 35 years who had been on ART for over a year and were willing to participate. Both virally suppressed and unsuppressed patients were included in the study. Severely ill patients and mental healthcare users were excluded from the study. Participants who met the inclusion criteria were identified at these clinics and invited to participate. Those willing to participate were given interview dates corresponding to their next appointment date.

### Data collection

Individual semi-structured interviews were held using an interview guide with open-ended questions. The interviews were conducted in Sepedi and English by a trained research assistant. The interviews took place from July 2023 to September 2023 and were audio recorded. The principal researcher was present during the interviews to take field notes.

### Data analysis

Recorded interviews were transcribed verbatim in Sepedi by the research assistant. The primary researcher and a Sepedi-speaking research assistant listened to the recorded interviews and compared them to the transcribed data, making corrections. The transcribed interviews were then translated into English by a language expert. Thematic analysis was conducted using a deductive coding process guided by the five thematic analysis stages: familiarisation, theme identification, indexing, charting mapping and interpretation.^15^ The principal researcher and the research assistant read through the transcripts several times, identifying concepts. All the concepts were coded and arranged into categories. The principal researcher and the assistant individually organised these into main themes and subthemes. Consensus was reached via discussion with a qualitative research expert (research supervisor). Eight themes with subthemes were identified.

Trustworthiness was enhanced through the following steps: audio-recording the interviews, prolonged engagement with the data and triangulation between the principal researcher, research assistant and research supervisor. A detailed description of the study setting assists with the transferability of the findings. Describing the steps of the research provides a clear audit trail for data methods.

### Ethical considerations

Ethical approval was obtained from the Turfloop Research and Ethics Committee (project number: TREC/31/2022:PG-) with permission from the Limpopo Provincial Department of Health research committee (LP_2023-06-011) and the Department of Health Mankweng Hospital Complex. Written consent was received from participants after they were informed verbally and in writing.

## Results

Fifteen young adults were invited to participate, 13 agreed to come and 12 patients signed informed consent forms. One patient came for his follow-up date but was no longer interested in participating. Among the 12 participants who agreed to participate in the individual interviews, nine were females, and three were males. Five were between 18 years and 29 years of age, and seven were from 30 years to 35 years. Four participants had unsuppressed viral load, while eight patients had suppressed viral load. Many of the participants were unemployed, with only three being employed. The characteristics of the 12 participants are presented in Table 1.

**TABLE 1:** Characteristics of the participants.

Participant	Age	Gender	Employment status	Viral load status	Treatment regimen
1	27	Female	Employed	Suppressed	First line
2	30	Female	Unemployed	Suppressed	First line
3	32	Female	Unemployed	Suppressed	First line
4	35	Male	Employed	Suppressed	First line
5	33	Female	Unemployed	Unsuppressed	First line
6	18	Female	Grade 11 student	Suppressed	First line
7	26	Female	College student	Suppressed	Second line
8	32	Female	Unemployed	Unsuppressed	Second line
9	21	Female	University student	Unsuppressed	Second line
10	29	Male	Employed	Suppressed	First line
11	24	Female	Unemployed	Suppressed	First line
12	35	Male	Unemployed	Unsuppressed	Second line

The authors identified eight major themes that participants perceived to have influenced their adherence to ART, namely: medication-related factors, healthcare system factors, healthcare workers’ attitudes, economic factors, disclosure, acceptance, mobile phone reminders and family support. Quotations supporting the themes and sub-themes are presented in Table 2. Data saturation was reached at the 10th interview as no new themes emerged from the 11th and 12th participant interviews.

**TABLE 2:** Major themes and sub-themes with supporting quotations.

Themes and sub-themes	Supporting quotations
**Theme 1 : Medication related factors**	
Side effects	‘Because of side effects I stopped for like five to six months … I started becoming swollen. I developed “tin-staff” [*Fungal infection*] like spots on my face and then I came back and continued with my treatment.’ (Participant 8, 32 years old, female unemployed, VL unsuppressed). ‘Initially when I was taking them, I had complications. I was becoming dizzy and also vomited. I came back here, then they gave me an explanation for such side-effects and was then given the peppermint ones which were then suitable for me.’ (Participant 7, 26 years old, female college student, VL suppressed). ‘I was unable to swallow the pill. Before taking it, they were crushing it for me so that I can take it in, but it was bitter as well.’ (Participant 6, 18 years old, female high school, VL suppressed).
Benefit of the single pill ART regimen	‘Of course, yes, because it’s not time and time again. You do not have to have it in the morning, during the day and in the evening. It is just once, then you relax. If you do not feel okay, you know you have your thing just at 08:00.’ (Participant 3, 32 years old, female, unemployed, VL suppressed).
Perceived benefits of ART	‘Yes, a lot because now I have gained weight, the time I wasn’t taking the medication I lost weight. I can even take this treatment again in a day if it was possible, I don’t want to get sick.’ (Participant 1, 27 years old, female, employed, VL suppressed). ‘It’s because when I take the medication I will live long, and I will have a longer life span and the virus will decrease in my body. They say If you don’t take medication you will end up becoming in a stage of AIDS.’ (Participant 11, 24 years old, female, unemployed, VL suppressed).
**Theme 2: Healthcare system factors**	
Availability of drugs	‘Like, there is no way you could come and find that pills are not available in between medication cycles.’ (Participant 5, 33 years old, female, unemployed, VL unsuppressed).
Long queues at clinics	‘The only thing is that they may be overcrowded the whole day, and you must come back by the following day…you have to que again, but it will be lesser and lesser than the previous days.’ (Participant 10, 29 years old, male, employed, VL suppressed).
Long travel distances to the clinic	‘It is quiet a distance … It affects me dearly, because at times I become penniless and then be forced to ask for help with money for transport.’ (Participant 5, 33 years old, female, unemployed, VL unsuppressed).
Referral procedure	‘Well, the problem is that I was not around, it’s the time I was working as a security, and I was moved to KwaZulu-Natal, and it happened that I didn’t take my particulars to collect medication when I moved. That side they refused to give me the medication because I didn’t have my green card to show the medication I was taking.’ (Participant 12, 35 years old, male, unemployed, VL unsuppressed).
**Theme 3: Attitudes of healthcare workers**	
Attitudes of healthcare workers	‘They are very good to me, not harassing us. Just when you are inside the consultation room, you become free to talk about your condition, and they give you the treatment. They do not say you need to queue again. Just where you are consulting from you get your medication.’ (Participant 2, 30 years old, female, unemployed, VL suppressed). ‘I like coming here because they treat us well unlike other clinics I went to before and they didn’t welcome me well because my baby was unwell. They asked me where I collect my medication and that was not in their books, so here they welcome people.’ (Participant 1, 27 years old, female, employed, VL suppressed).
Negative attitudes	‘Eish at the local clinic where I use to go and collect medicine, I ended up changing clinics because there was a lady who is a home-based care giver who was talking bad about our status. When we are collecting our treatment for HIV, she would talk about us saying we didn’t collect the medication, she would say this person and that person don’t just see them they have a disease. That made many people uncomfortable and open a case against her.’ (Participant 12, 35 years old, male, unemployed, VL unsuppressed).
**Theme 4: Economic factors**	
	‘Sometimes you find that there will be no food, and I will become scared to take my tablets on an empty stomach. As I have indicated earlier on when I said there are times when I will skip medication because of non-availability of food, I am not too sure whether the clinic has something like food parcels to assist.’ (Participant 5, 33 years old, female, unemployed, VL unsuppressed). ‘Transport affects me dearly, because at times I become penniless and then be forced to ask for help with money for transport.’ (Participant 5, 33 years old, female, unemployed, VL unsuppressed).
**Theme 5: Disclosure**	
Benefits of disclosure	‘It’s a relief because it takes away the fear in you. Of course, at the beginning it is scarry to disclose because you will feel like they are going to isolate you all. So, it is quite a relief for people to know what one is going through.’ (Participant 6, 18 years old, female, high school, VL suppressed). ‘When I meet a person who is interested in me, I will tell him of my situation so that tomorrow if he falls sick and should not blame me that I have infected him.’ (Participant 2, 30 years old, female, unemployed, VL suppressed).
Negative side of disclosure	‘There were many judgements, I even stopped again for six months … they would say you cannot touch whatever belongs to me when you are HIV positive. You do not love your child; you are going to die just like your mother.’ (Participant 8, 32 years old, female unemployed, VL unsuppressed).
**Theme 6: Acceptance**	
	‘I could blame myself I can say it was my carelessness because I could not take care of myself as I was still innocent. I was shocked, a bit scared and feeling hurt, but it was just to accept that that is how life is. You will only ask yourself as to where did you get infected and by who. Such things that you may not even have answers to. It is just a matter of saying: “Accept and go on with your life” I then made peace with it and continued with my life, became okay and then told myself that I must take the treatment.’ (Participant 3, 32 years old, female, unemployed, VL suppressed) ‘Well, I was disappointed, but I told myself it’s not the end of the world, I blamed myself here and there and eventually I accepted.’ (Participant 4, 34 years old, male, employed, VL suppressed).
**Theme 7: Mobile phone reminders**	
	‘I set an Alarm, 08:00 I make sure by that time I am at home, and I have eaten then I drink the medication.’ (Participant 6, 18 years old, female, high school student, VL suppressed). ‘When the alarm starts ringing, they will say, mom it is that time of medication. They call them ‘sweets’. They will say it is time and there are your sweets. Even when I am coming to the clinic, I inform them and they will remind me when the time arrives, so I will go. That is the encouragement I get from the home.’ (Participant 2, 30 years old, female, unemployed, VL suppressed).
**Theme 8: Family support**	
Good support	‘The thing is, because my mother passed away from HIV, when the news came in that am also HIV positive, it was not a shock. So, it was just supporting all the way. I have my entire family supportive; l also have my father and my sister.’ (Participant 10, 29 years old, employed, VL suppressed). ‘My sister encouraged me to take the medication and that there are a lot of people who are taking this medication. She knows this lady whose been having HIV for more than 20 years and she still lives so it’s not the end of the world.’ (Participant 4, 34 years old, male, employed, VL suppressed).
Lack of support	‘I left school in Grade 11, when my mother passed away. I started having challenges. … I had no choice but to fend for myself. No sister, no brother or anyone to take care of me. My father was there, but extremely into ladies. He never thought of being there for his children.’ (Participant 3, 32 years old, female, unemployed, VL suppressed).

ART, antiretroviral therapy; HIV, human immunodeficiency viruses; AIDS, acquired immunodeficiency syndrome; VL viral load.

Medication-related factors such as side effects, the size of the pill and its bitter taste discouraged participants from taking the medication regularly. The more recent single-pill regimen assists adherence as patients do not need to take multiple pills. The perceived benefits of ART positively influenced ART adherence. Health system factors that influenced participants were long clinic waiting times and long distances to travel for medication. One participant moved to another province without a referral note and could subsequently not access his medication. Positive attitudes of healthcare workers and counselling encouraged participants to take their medication regularly. However, one participant had a bad experience at the clinic when collecting his medication when he felt his privacy was not respected and that negatively affected his chances to remain in care and poorly affected his adherence to ART. Economic factors such as not having money for food and transport to the clinic contributed to poor adherence. Some patients are scared to take pills without eating as they feel the side effects may be worse.

Patients who felt they would be stigmatised feared disclosing their HIV status. Fear of disclosure results in patients defaulting on treatment as they do not want to be caught taking the ART medication; however, in this study, disclosure assisted them in acquiring needed support. Participants who accepted the condition found it easy to adhere to medication, while those who struggled to accept it blamed themselves for contracting the disease.

Many participants set reminders on their mobile phones to remember to take their medication at the prescribed time, which facilitated their ART adherence. Most participants in this study were encouraged to take medication by their family, children or siblings, and this family support played a significant role in their adherence to ART medication.

## Discussion

This study identified several factors that influence ART adherence in young adults in the Limpopo province of South Africa. These included medication-related factors, healthcare system factors, attitudes of healthcare workers, economic factors, disclosure, acceptance, mobile phone reminders and family support.

### Medication-related factors

Several of the participants stated that side effects negatively influenced their ART adherence, which led to some participants discontinuing ART medication. This concurred with the literature that reported participants discontinuing ART medication because of intolerance of the side effects.^5,6^ Nevertheless, other patients found ways to adjust to treatment by either taking medication when going to bed or crushing the medication rather than stopping ART, which is also supported by other studies.^7,9,16^ The participants acknowledged that knowing the benefits of ART helped them continue their treatment. The benefits of ART are widely acknowledged to be a catalyst for adherence as also stated by the participants.^16,17,18,19^ The single-pill ART regimens are associated with better adherence, as also mentioned by the participants and similarly supported by the literature.^7,18,20^

### Healthcare system factors

The participants noted the constant availability of ART at clinics as a facilitator of adherence, which is a similar finding in other studies.^19^ Other participants encountered challenges such as long clinic queues and travelling long distances to the clinic. These challenges lead to patient dissatisfaction and poor adherence to ART treatment. This finding is consistent with other studies, which found long queues at the facilities and slow service are associated with poor retention in care and consequently lead to poor adherence to ART.^8,21,22^

### Attitudes of healthcare workers

Many participants experienced positive attitudes with healthcare workers (HCWs) at clinics, encouraging them to adhere to their ART medication. Similarly, other studies have also found that positive relationships increase patient confidence and motivation to remain on ART.^8,10,23^ Some participants experienced bad attitudes from the healthcare professionals where they felt that their confidentiality was breached and HCWs did not properly communicate with them. This led to changing clinics and poor adherence to ART. Similarly, several studies found that bad HCW attitudes are associated with patients’ dissatisfaction, poor retention of care and poor adherence to ART medication.^8,9,20,21^

### Economic factors

Economic factors such as a lack of money for transport and food availability were also found to influence adherence to ART. Several studies also found economic factors as a significant social determinant of HIV and/or AIDS and access to ART treatment.^7,8,19,23,24^

### Disclosure

Some participants did not disclose their HIV status because of fear of discrimination. This is similar in both developed and developing countries where patients fear disclosing their status for fear of stigmatisation, and this robs them of a chance to be supported in taking their ART.^6,22,25^ In some communities, HIV and/or AIDS is still a taboo subject, and, as such, families would advise the person with HIV not to disclose their condition to others.^7^ Stigma discourages patients from taking their ART medication out of fear of being noticed, thus affecting their adherence.^7,18,23^ On the other hand, this study found that the disclosure of HIV status encouraged family support and acceptance, which facilitated adherence to ART.

### Acceptance

Acceptance of HIV status was also found to facilitate ART adherence, whereas denial and self-blame were associated with discontinuation of treatment. This finding is echoed in the literature finding acceptance of HIV as an important factor in ART adherence.^13,26,27^

### Mobile phone reminders

Setting reminders was identified as a facilitator of ART adherence. Many of the participants used their mobile phones to remind them to take their medication at the prescribed time. The use of devices to set reminders has also been reported in the literature on interventions used to improve ART adherence.^28,29,30^

### Family support

The participants in this study indicated that family support played a significant role in ART adherence. This is also supported by the literature.^30,31^ Healthy family relationships enhance disclosure and acceptance, encouraging adherence to ART medication.^26,27,32,33,34,35,36^ For this reason, family support is encouraged in the latest ART clinical guidelines.^4^ Unfortunately, no clear interventions have been suggested in the South African National ART programme to strengthen family support. Clearly defined interventions, such as a routine family conference or couple counselling, should be encouraged to harness and strengthen family support. This has huge potential to improve adherence to the national ART programme.

### Limitations

Participants may not have responded truthfully to questions for fear that they would not be treated the same way after the interview if they criticised the service. The authors tried to minimise this by clearly explaining the research and reassuring the participants that their responses would not affect their treatment at the clinics.

## Conclusion

This study highlights the complex interplay of factors influencing ART adherence, ranging from medication-related factors, healthcare system factors, attitudes of healthcare workers, economic factors, disclosure, acceptance, mobile phone reminders and having family support. Having family support was a factor that was identified to positively influence ART adherence as it leads to disclosure and acceptance of the disease, better emotional well-being and subsequently improved ART adherence. As healthcare providers, we should work on promoting acceptance and disclosure of HIV status and strengthening family support for people living with HIV. It is encouraging to note that many patients are aware of and have recognised the benefits of ART, which motivates them to adhere to treatment. Further research into family-oriented interventions is recommended. Implementation of strategies to strengthen family support has the potential to improve ART adherence, better treatment outcomes for individuals living with HIV and the possibility of achieving the UNAIDS 95-95-95 targeted goal.
